# *SNAC3* Transcription Factor Enhances Arsenic Stress Tolerance and Grain Yield in Rice (*Oryza sativa* L.) through Regulating Physio-Biochemical Mechanisms, Stress-Responsive Genes, and Cryptochrome 1b

**DOI:** 10.3390/plants12142731

**Published:** 2023-07-23

**Authors:** Marootpong Pooam, Enas M. El-Ballat, Nathalie Jourdan, Hayssam M. Ali, Christophe Hano, Margaret Ahmad, Mohamed A. El-Esawi

**Affiliations:** 1 UMR CNRS 8256 (B2A), IBPS, Sorbonne Université, 75005 Paris, France; 2 Botany Department, Faculty of Science, Tanta University, Tanta 31527, Egypt; 3 Department of Botany and Microbiology, College of Science, King Saud University, Riyadh 11451, Saudi Arabia; 4 Laboratoire de Biologie des Ligneux et des Grandes Cultures, INRAE USC1328, Campus Eure et Loir, Orleans University, 45067 Orleans, France

**Keywords:** antioxidants, arsenic stress, gene expression, osmolytes, *SNAC3*, transgenic rice

## Abstract

Arsenic (As) is one of the toxic heavy metal pollutants found in the environment. An excess of As poses serious threats to plants and diminishes their growth and productivity. NAC transcription factors revealed a pivotal role in enhancing crops tolerance to different environmental stresses. The present study investigated, for the first time, the functional role of *SNAC3* in boosting As stress tolerance and grain productivity in rice (*Oryza sativa* L.). Two *SNAC3*-overexpressing (*SNAC3*-OX) and two *SNAC3*-RNAi transgenic lines were created and validated. The wild-type and transgenic rice plants were exposed to different As stress levels (0, 25, and 50 µM). The results revealed that *SNAC3* overexpression significantly improved rice tolerance to As stress and boosted grain yield traits. Under both levels of As stress (25 and 50 µM), *SNAC3*-OX rice lines exhibited significantly lower levels of oxidative stress biomarkers and *OsCRY1b* (*cryptochrome 1b*) expression, but they revealed increased levels of gas exchange characters, chlorophyll, osmolytes (soluble sugars, proteins, proline, phenols, and flavonoids), antioxidant enzymes (SOD, CAT, APX, and POD), and stress-tolerant genes expression (*OsSOD-Cu/Zn*, *OsCATA*, *OsCATB*, *OsAPX2*, *OsLEA3*, *OsDREB2B, OsDREB2A*, *OsSNAC2*, and *OsSNAC1*) in comparison to wild-type plants. By contrast, *SNAC3* suppression (RNAi) reduced grain yield components and reversed the aforementioned measured physio-biochemical and molecular traits. Taken together, this study is the first to demonstrate that *SNAC3* plays a vital role in boosting As stress resistance and grain productivity in rice through modulating antioxidants, photosynthesis, osmolyte accumulation, and stress-related genes expression, and may be a useful candidate for further genetic enhancement of stress resistance in many crops.

## 1. Introduction

Plants often encounter various environmental stresses throughout their lifetime, such as heavy metals toxicity, salinity, and drought [1,2]. Among the extremely toxic heavy metal environmental pollutants, arsenic (As) is present in water and agricultural soils due to different anthropogenic activities [2,3]. An excess of As causes critical threats to plant, animal, and human health [2]. As toxicity drastically diminishes crop growth, development, metabolism, and yield through influencing the physiological and biochemical pathways and inducing generation of toxic reactive oxygen species (ROS) [4,5]. To combat such stress-induced adverse effects, plants employ a variety of complex processes at different levels, including accumulation of osmolytes, binding metallothionein, sequestration of metals into vacuoles, and induction of several antioxidant enzymes and stress-responsive genes [5,6,7].

The genetic engineering approach has also proved efficient for developing plant varieties with enhanced tolerance to environmental stresses. Over the last years, various transcription factor family members such as NAC (NAM, ATAF1/2, and CUC2), bHLH, AP2/ERF, WRKY, MYB, CAMTA, bZIP, DREB, and NF-Y have revealed remarkable functions in mediating plant resistance to adverse stresses [1,8,9,10,11,12,13,14,15,16]. The name “NAC” is derived from the initials of the three founding members of this transcription factor family: NAM, ATAF1/2 (*Arabidopsis* Transcription Activation Factor 1/2), and CUC2. The NAC transcription factor family regulates diverse developmental processes and stress responses in plants. NAC proteins are distinguished by remarkably maintained DNA-binding domains, recognized as NAC domains, and have demonstrated a fundamental role in the regulation of plant cell division, organ development, senescence, iron homeostasis, and environmental stress responsiveness [9,17,18,19,20]. Overexpression of *ANAC072*, *ANAC055*, or *ANAC019* stimulated by ABA, salt, and drought stresses enhances transgenic *Arabidopsis* tolerance to drought stress [21]. Additionally, *ANAC013* is a membrane-linked NAC protein that boosted transgenic *Arabidopsis* resistance to oxidative stress [20,22]. Another NAC factor, *AtJUB1*, mediated plant growth through suppressing the *GA3ox1* and *DWF4* genes, encoding brassinosteroids and gibberellic acid biosynthesis enzymes, as well as via inducing *DELLA* genes [1,23,24]. Moreover, *Arabidopsis* overexpressing *AtJUB1* or banana overexpressing *MusaNAC042* (*AtJUB1* homologue) displayed increased resistance to multiple environmental stresses including excessive salinity and drought, while *Arabidopsis AtJUB1* knockdown or tomato *SlJUB1* silencing mitigated resistance to oxidative and drought stresses [25,26,27,28]. In the regulation of plant responsiveness to abiotic stress conditions, the expression of *SlJUB1* and *AtJUB1* can be promptly linked to *SlDREB1*, *SlDREB2*, and *AtDREB2A* promoters, respectively, which encode essential abiotic stress regulators, including AP2-type TFs [1,25,29]. Moreover, *AtJUB1* could function downstream of *AtHB13*, which positively regulates drought stress tolerance [1,26]. Chen et al. [12] stated that *ZmNAC2*, a transcription factor from maize, could augment osmotic stress resistance in transgenic *Arabidopsis*. *IPA1* also enhanced drought stress tolerance through inducing *SNAC1* in rice [13]. Moreover, *AhMYB30* boosted freezing and salt resistance in *Arabidopsis* [14]. *AtMYB40* transcription factor also displayed a functional role in As tolerance in *Arabidopsis* [30]. Furthermore, *OsARM1* overexpression enhanced rice sensitivity to As stress [31].

Rice (*Oryza sativa* L.) is amongst the foremost economically crucial cereal crops globally and supplies core food crops for inhabitants worldwide [20]. Environmental stresses negatively influence rice yield and grain quality. Moreover, rice retains the maximum level of As amongst all other crops because of the existence of aquaglyceroporins and phosphate transporters [2]. More importantly, due to the severe impacts of climate change and growing global population, there is an urgent requirement to develop more stress-resistant rice cultivars for use in breeding programs to boost crop yields to satisfy global food demands. Over the past decades, different rice varieties have been genetically engineered for enhanced stress tolerance via overexpressing several stress-related transcription factors, including NACs. For example, transgenic rice lines overexpressing *SNAC2* displayed considerably increased resistance to salt and cold stresses [32], while the overexpression of the root-specific *OsNAC9*, *OsNAC6*, or *OsNAC10* significantly augmented tolerance to drought in rice [33,34]. Overexpression of *ONAC066* improved drought and oxidative stress resistance in rice [1]. *ONAC095* plays different functional roles in cold- and drought stress responsiveness [35]. It negatively regulates drought responses but positively regulates cold responses in rice plants [35]. More importantly, overexpression of some stress-responsive NAC transcription factors significantly augmented rice resistance to severe environmental stress conditions without causing adverse impacts on crop yields [1,33,36,37], indicating the importance of the use of those NAC genes for further enhancement of stress tolerance and crop yield [38,39]. For example, transgenic rice plants overexpressing *OsNAC5*, *OsNAC10,* and *SNAC1* and subjected to drought stress conditions displayed increased root diameter, drought tolerance, and grain yield [20,36,40,41].

Another rice NAC transcription factor, *SNAC3*, has been previously documented as a significant regulator of drought, heat, and oxidative stress responsiveness in rice plants via modulating abscisic acid–independent pathways and ROS [20]. However, the function of *SNAC3* in mediating As stress tolerance and rice grain yield attributes has not been studied and validated yet. Therefore, the present study aimed to investigate whether *SNAC3* has a vital role in augmenting rice tolerance to As stress and improving grain yield. *SNAC3*-overexpressing and *SNAC3*-RNAi transgenic rice lines were created utilizing an *Agrobacterium*-mediated transformation method. Various morpho-physiological, biochemical and molecular parameters as well as grain yield traits have been analyzed and recorded to reveal the significant differences between transgenics and wild-type rice plants in the current investigation.

## 2. Results and Discussion

### 2.1. Generation and Molecular Characterization of Transgenic Rice Lines

**Figure 1:**
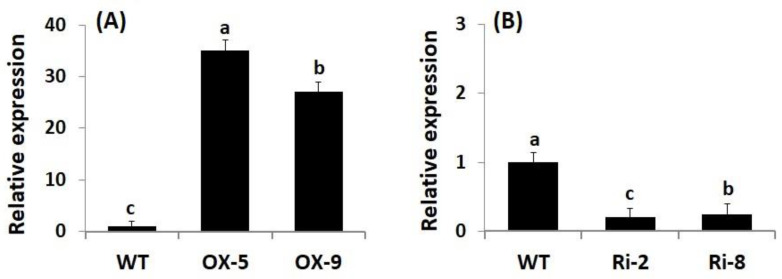
Characterization of transgenic rice lines. (**A**) Expression of *SNAC3* in *SNAC3*-overexpressing lines; and (**B**) expression of *SNAC3* in *SNAC3*-RNAi lines. Data are means ± SE (*n* = 5). Different letters above bars (a, b, c) indicate a significant difference among rice lines (*p* ≤ 0.05).

Genetic engineering has emerged as a promising approach for boosting crop stress resistance and accelerating plant breeding programs. To evaluate whether *SNAC3* contributes significantly to enhancing rice resistance to As stress and boosting grain yield, *SNAC3*-OX and *SNAC3*-RNAi T_3_ transgenic rice lines were developed utilizing the *Agrobacterium*-mediated transformation method in the current study. qRT-PCR analysis indicated that the expression level of *SNAC3* in *SNAC3*-OX lines, OX-5 and OX-9, were 34.9- and 27.1-fold higher, respectively, than that in wild-type (WT) plants (Figure 1A), whereas the expression level in *SNAC3*-RNAi lines, Ri-2 and Ri-8, were calculated to be 20 and 25% of the level in WT plants (Figure 1B), respectively. These transgenic lines were selected for use in the As stress experiment and for subsequent morpho-physio-biochemical and transcriptional analyses of the present study.

### 2.2. SNAC3 Overexpression Enhances While SNAC3 Suppression Reduces Survival Rate, Leaf Relative Water Content, and Growth Characteristics of Rice under Arsenic Stress

**Figure 2:**
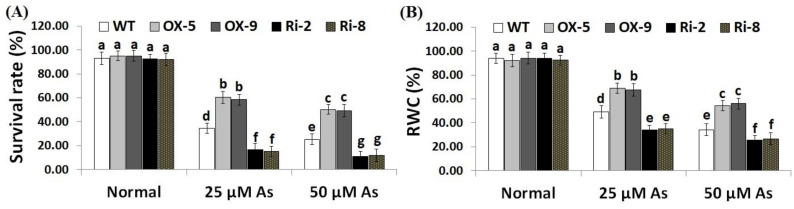
Survival rate (**A**) and RWC (**B**) of the wild type (WT) and transformed lines subjected to varying arsenic doses for 12 days. Values represent means ± SE (*n* = 5). Different letters above bars represent a significant difference among lines (*p* ≤ 0.05).

To explore whether *SNAC3* could contribute to As stress resistance, a 10-day recovery period was applied after As stress treatment to record plants’ survival rate. The results showed that under both doses of As stress (25 and 50 µM), the survival rate of *SNAC3*-OX rice lines (OX-5 and OX-9) was remarkably higher than that of the wild-type plants, while *SNAC3*-RNAi lines (Ri-2 and Ri-8) exhibited significantly lower survival rates comparing to non-transformed plants (Figure 2A). In addition, leaf relative water content (RWC) represents one of the main indicators of water level balancing in plants. RWC was therefore estimated in the leafy tissues of the non-transgenic, *SNAC3*-OX, and *SNAC3*-RNAi plants. The findings indicate that both levels of As stress (25 and 50 µM) significantly reduced the RWC of non-transgenic and transgenic rice plants comparing to those grown under optimal growth conditions (Figure 2B). Moreover, RWC of As stress-treated *SNAC3*-OX plants was noticeably higher than that of As stress-treated non-transgenic plants, while As stress-treated *SNAC3*-RNAi plants had significantly lower RWC, comparing to As stress-treated non-transgenic plants. These results suggest that *SNAC3* overexpression enhances As stress resistance in transgenic rice plants via maintaining the water status of plants. Our results agree with those demonstrated by Fang et al. [20], who revealed the enhanced survival rate of *SNAC3*-overexpressing rice plants under drought circumstances. Similarly, Yuan et al. [1] reported enhanced survival rate and RWC of *ONAC066*-overexpressing rice plants cultivated under drought circumstances.

To further validate the function of *SNAC3* in As stress tolerance, plant growth traits were measured. The findings demonstrated that both of As stress concentrations (25 and 50 µM) significantly restricted plant height, fresh weights of root and shoot, and dry weights of root and shoot of the non-transgenic and transgenic rice plants, in comparison to those grown under normal circumstances (Table 1). However, no significant differences in these growth parameters were detected between the non-transgenic and transgenic plants grown under optimal (0 As) treatment. On the other hand, under both levels of As stress (25 and 50 µM), all growth parameters of *SNAC3*-OX rice lines (OX-5 and OX-9) were remarkably higher than that of the wild-type plants, while *SNAC3*-RNAi lines (Ri-2 and Ri-8) exhibited significantly reduced growth parameters, comparing to the non-transgenic plants (Table 1). Such results further indicate that *SNAC3* has a crucial function in rice As stress resistance through improving plant growth characteristics. Our results agree with that of Fang et al. [20], who recorded enhanced plant height of *SNAC3*-overexpressing transgenic rice plants under drought circumstances. Additionally, Yuan et al. [1] reported augmented performance of *ONAC066*-overexpressing rice plants grown under drought circumstances.

### 2.3. Overexpression of SNAC3 Reduces While Suppression of SNAC3 Increases Arsentic Uptake and Oxidative Stress Biomarkers in Rice under Arsenic Stress

**Figure 3:**
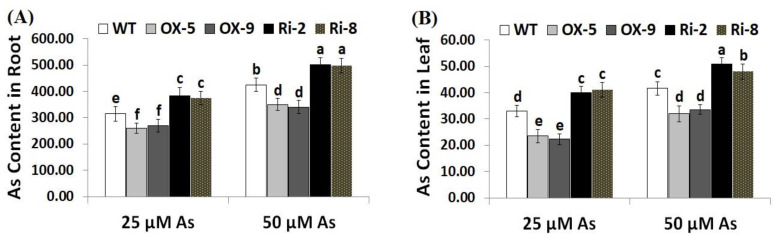
Arsenic concentration (µg g^−1^ DW) in the roots (**A**) and leaves (**B**) of the wild type (WT) and transformed lines under varying arsenic doses for 12 days. Values represent means ± SE *(n* = 5). Different letters above bars represent a significant difference among lines (*p* ≤ 0.05).

Arsenic concentration was assessed in the roots and leaves of the wild-type, *SNAC3*-OX, and *SNAC3*-RNAi plants. The results revealed that As concentration was higher in the plant root than shoot (Figure 3A,B). Moreover, As uptake increased in the roots and shoots of rice plants with increasing As stress concentration. On the other hand, the roots of *SNAC3*-OX rice lines, OX-5 and OX-9, exhibited significant reductions in As content by 17.5 and 14.6% at 25 µM As, respectively, and by 17.6 and 20% at 50 µM As, respectively, while the roots of *SNAC3*-RNAi lines, Ri-2 and Ri-8, had significant increases in As by 21.6 and 18.7% at 25 µM As, respectively, and by 18.1 and 17.2% at 50 µM As, respectively, as compared to As stress-treated wild-type plants (Figure 3A). Moreover, the leaves of *SNAC3*-OX rice lines, OX-5 and OX-9, exhibited significant reductions in As content by 28.8 and 32.4% at 25 µM As, respectively, and by 23.1 and 19.5% at 50 µM As, respectively, while the leaves of *SNAC3*-RNAi lines, Ri-2 and Ri-8, exhibited significant increases in As by 21.2 and 24.2% at 25 µM As, respectively, and by 22.6 and 15.4% at 50 µM As, respectively, as compared to As stress-treated wild-type plants (Figure 3B). The results indicate that the *SNAC3* gene contributes to mitigating As toxicity in rice plants grown under As stress circumstances via reducing As uptake. Similarly, *WRKY6* transcription factor conferred As stress tolerance in *Arabidopsis* via restricting As uptake [42].

**Figure 4:**
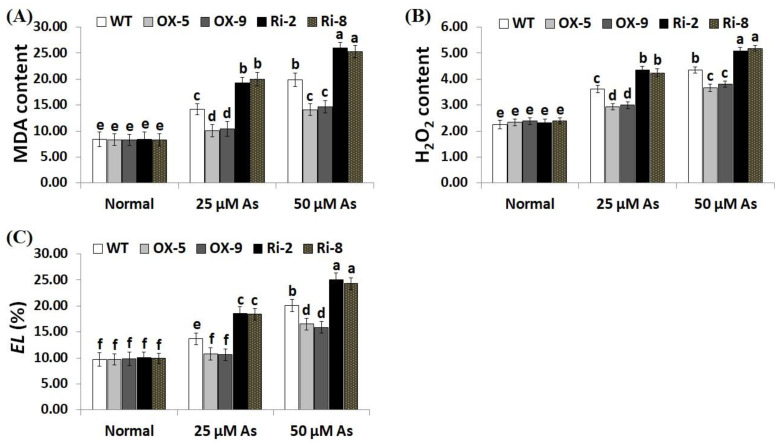
Levels of lipid peroxidation (MDA, µM g^−1^ FW) (**A**), H_2_O_2_ (µM g^−1^ FW) (**B**), and electrolyte leakage (*EL*) (**C**) of the non-transformed (WT) and transformed rice lines under varying arsenic doses for 12 days. Values represent means ± SE (*n* = 5). Different letters above bars represent a significant difference among lines (*p* ≤ 0.05).

Overproduction of ROS causes toxic effects and destruction to plant cells. Electrolyte leakage (*EL*) represents a main indicator for the damage of the cell membrane [43]. Moreover, MDA is a key indicator for the free radicals-induced damage of lipid peroxidation end products [44]. Consequently, developing more stress-resistant crop cultivars is urgently required. To explore whether *SNAC3*-OX lines scavenge ROS, levels of oxidative stress markers (MDA, H_2_O_2_, and *EL*) were measured in the leaves of transformed and non-transformed plants under optimal and As treatments. As stress concentrations (25 and 50 µM) significantly enhanced the level of oxidative stress markers of the transgenic and non-transgenic rice plants, in comparison with those grown under optimal growth circumstances (Figure 4A–C). However, no considerable variances were recorded in MDA, H_2_O_2_, and *EL* between the transgenic and non-transgenic plants under standard conditions. In contrast, under both concentrations of As stress, levels of oxidative stress markers of the two *SNAC3*-OX lines were considerably lower than those of the non-transgenic plants, while the two *SNAC3*-RNAi lines demonstrated considerably higher level of MDA, H_2_O_2_, and *EL*, comparing to non-transgenic plants (Figure 4A–C). These findings indicate that *SNAC3* overexpression in rice plants counteracts the toxic impacts of ROS accumulation, mitigates the oxidative damage, maintains a lower degree of cell membrane lipid peroxidation, and confers enhanced tolerance to As stress. Our results agree with that demonstrated by Fang et al. [20], who demonstrated reduced H_2_O_2_ levels in *SNAC3*-overexpressing transgenic rice plants under heat stress circumstances. Yuan et al. [1] also recorded reduced H_2_O_2_ content in *ONAC066*-overexpressing rice plants grown under drought stress conditions. *WRKY6* transcription factor also reduced the oxidative stress level and conferred As stress tolerance in *Arabidopsis* [42].

### 2.4. Overexpression of SNAC3 Enhances While Suppression of SNAC3 Reduces Chlorophyll Content, Gas-Exchange Parameters, and Osmolyte Accumulation in Rice under Arsenic Stress

**Figure 5:**
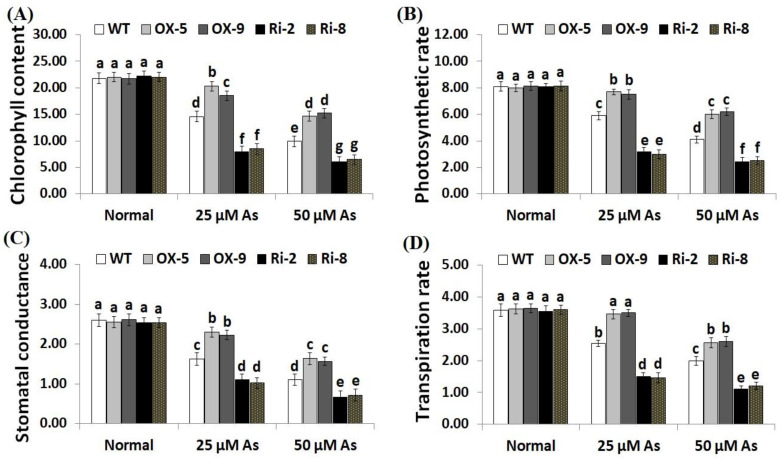
Chlorophyll content (mg g^−1^ FW) (**A**), photosynthesis rate (*P_n_*, μmol m^2^s^−1^) (**B**), stomatal conductance (*g_s_*, mol m^2^s^−1^) (**C**), and transpiration rate (*E*, mmol m^2^s^−1^) (**D**) of the non-transformed (WT) and transformed rice lines under varying arsenic doses for 12 days. Values represent means ± SE (*n* = 5). Different letters above bars represent a significant difference among lines (*p* ≤ 0.05).

Environmental factors have adverse impacts on chlorophyll and gas exchange attributes in plants. To investigate whether *SNAC3* could improve chlorophyll concentration and gas-exchange traits in rice plants grown under As stress, we measured the chlorophyll content, photosynthesis rate (*P_n_*), stomatal conductance (*g_s_*) and transpiration rate (*E*) in the non-transformed, *SNAC3*-OX, and *SNAC3*-RNAi plants grown under normal conditions and both levels of As stress (25 and 50 µM). The findings indicate that both concentrations of As stress significantly decreased the rates of chlorophyll and gas-exchange parameters of the non-transformed and transformed rice plants in comparison to those grown under standard growth circumstances (Figure 5A–D). Conversely, under standard circumstances, no significant variations were detected in chlorophyll content and gas-exchange parameters between the non-transformed and transformed lines. In contrast, under both concentrations of As stress, levels of chlorophyll, *P_n_*, *g_s_*, and *E* of the two *SNAC3*-OX lines were noticeably greater than those of the non-transgenic plants, while the two *SNAC3*-RNAi lines showed markedly lower levels of chlorophyll and gas-exchange parameters, comparing to the non-transformed plants (Figure 5A–D). These findings indicate that *SNAC3* overexpression enhances As stress resistance through boosting photosynthesis and gas-exchange processes in rice leaves. Similarly, Yuan et al. [1] reported improved chlorophyll concentration in *ONAC066*-overexpressing rice plants cultivated under stress circumstances.

**Figure 6:**
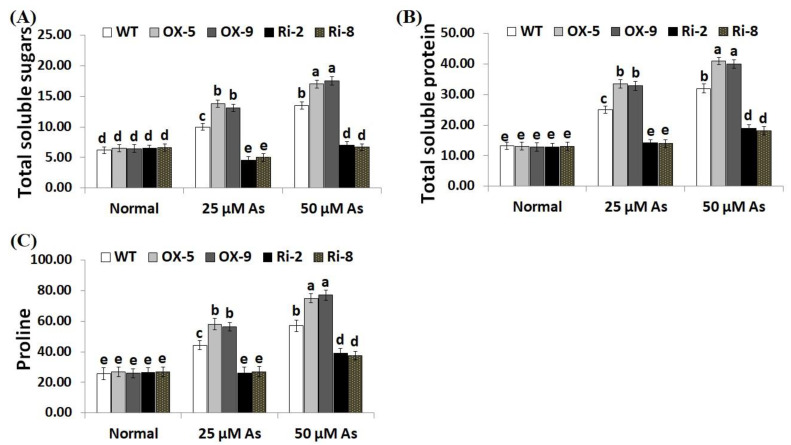
Contents of total soluble sugars (mg g^−1^ FW) (**A**), total soluble proteins (mg g^−1^ FW) (**B**), and proline (µg g^−1^ FW) (**C**) of the wild-type (WT) and transformed rice lines under varying arsenic doses for 12 days. Values represent means ± SE (*n* = 5). Different letters above bars represent a significant difference among lines (*p* ≤ 0.05).

**Figure 7:**
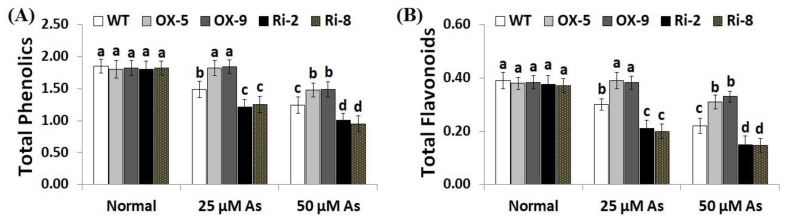
The total phenolics (mg gallic acid/g extract) (**A**) and total flavonoids (mg catechin/g extract) (**B**) of the wild-type (WT) and transformed rice lines under varying arsenic doses for 12 days. Values represent means ± SE (*n* = 5). Different letters above bars represent a significant difference among lines (*p* ≤ 0.05).

Soluble sugars, proline, proteins, phenolics, flavonoids, and other compatible solutes are useful osmoprotectants for plants under stress circumstances [45,46]. Soluble sugars and proteins also induce the efficiency of plant cells to resist dehydration and maintain biological molecules’ functions [47]. To dissect the mechanistic roles of *SNAC3* in As stress tolerance and osmoregulation process, we estimated the contents of soluble proteins, sugars, proline, total phenolics, and total flavonoids in the non-transformed and transformed rice lines grown under As treatments. The results revealed that no noticeable variations were recorded in the level of sugars, soluble proteins, proline, total phenolics, and total flavonoids between the transformed and non-transformed plants grown under optimal circumstances (Figure 6A–C; Figure 7A,B). However, under both concentrations of As stress, level of soluble sugars, proteins, proline, total phenolics, and total flavonoids of the two *SNAC3*-OX lines were considerably higher than those of the non-transformed plants, while the two *SNAC3*-RNAi lines exhibited significantly lower levels of soluble sugars, soluble proteins, proline, phenolics, and flavonoids, comparing to the non-transgenic plants (Figure 6A–C; Figure 7A,B). These findings revealed that *SNAC3* overexpression enhanced As stress tolerance in rice plants through increasing osmolyte accumulation in plant cells. Similarly, Yuan et al. [1] revealed enhanced synthesis of proline and soluble sugars in *ONAC066*-overexpressing rice plants grown under drought circumstances.

### 2.5. Overexpression of SNAC3 Induces While Suppression of SNAC3 Reduces Antioxidant Enzyme Activities in Rice under Arsenic Stress

**Figure 8:**
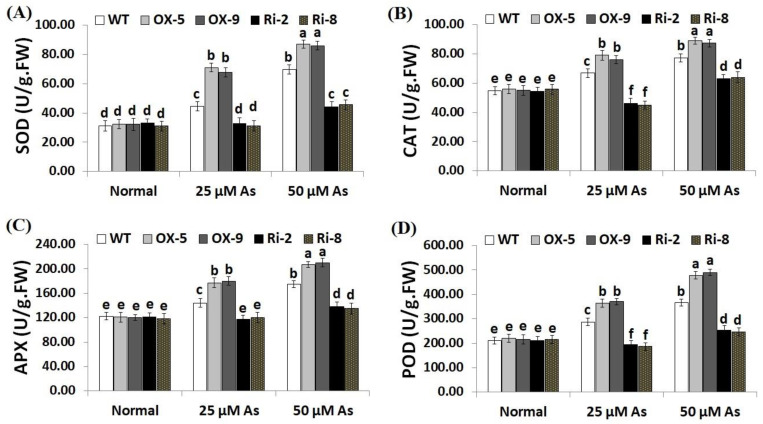
Levels of enzymic antioxidants ((**A**): SOD, (**B**): CAT, (**C**): APX and (**D**): POD) of the wild-type (WT) and transformed rice lines under varying arsenic doses for 12 days. Values represent means ± SE (*n* = 5). Different letters above bars represent a significant difference among lines (*p* ≤ 0.05).

Enzymic antioxidants have a crucial role in ROS detoxification and thus boosting plant ability to tolerate stresses [48]. CAT assists in the detoxification of ROS under stress, while APX assists in the fine-tuning of ROS signals [20,49]. We therefore measured the activities of SOD, CAT, POD and APX in the non-transgenic and transgenic rice plants grown under As stress circumstances (Figure 8A–D). The results revealed no obvious differences in the enzymic antioxidant levels among the transgenic and non-transgenic lines grown under optimal circumstances (Figure 8A–D). Conversely, under both concentrations of As stress (25 and 50 µM), the levels of SOD, CAT, POD and APX of the two *SNAC3*-OX lines were considerably higher than those of the non-transformed plants, while the two *SNAC3*-RNAi lines showed significantly lower levels of SOD, CAT, APX, and POD in comparison with the non-transgenic plants (Figure 8A–D). These findings indicate that *SNAC3* overexpression induces the enzymic antioxidant levels of transgenic rice lines which in turn scavenge ROS, causing decreased oxidative stress and enhanced As stress tolerance.

### 2.6. SNAC3 Overexpression and Suppression Modulate Abiotic Stress-Associated Genes Expression in Rice under Arsenic Stress

**Figure 9:**
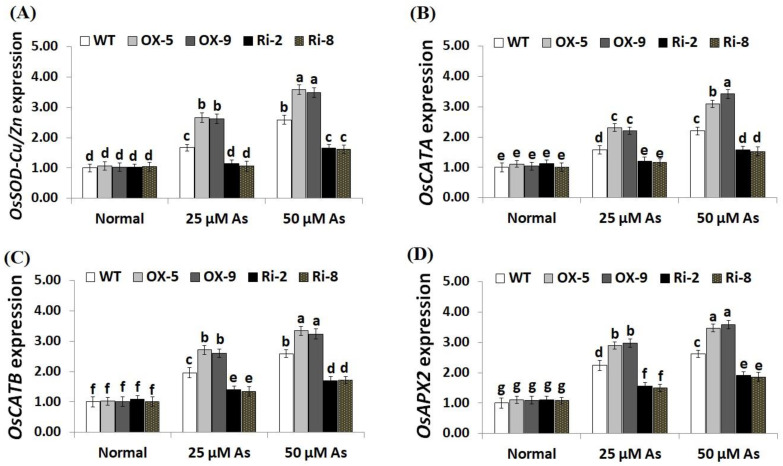
Expression of *OsSOD-Cu/Zn* (**A**), *OsCATA* (**B**), *OsCATB* (**C**), and *OsAPX2* (**D**) genes of the wild-type (WT) and transgenic rice lines under varying arsenic doses for 12 days. Values represent means ± SE (*n* = 5). Different letters above bars represent a significant difference among lines (*p* ≤ 0.05).

**Figure 10:**
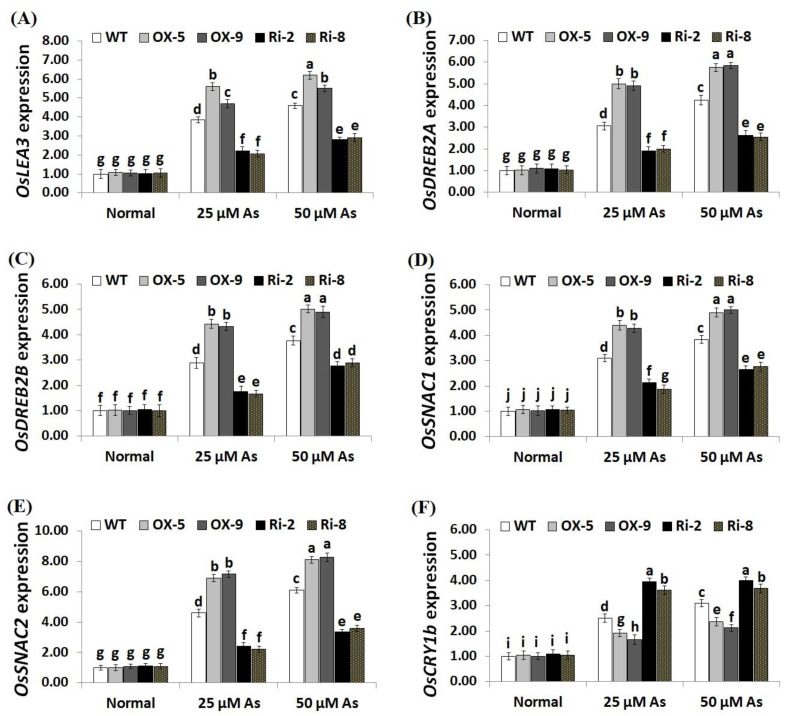
Expression of *OsLEA3* (**A**), *OsDREB2A* (**B**), *OsDREB2B* (**C**)*, OsSNAC1* (**D**), *OsSNAC2* (**E**), and *OsCRY1b* (**F**) genes of the wild-type (WT) and transgenic rice lines under varying arsenic doses for 12 days. Values represent means ± SE (*n* = 5). Different letters above bars represent a significant difference among lines (*p* ≤ 0.05).

To unravel the downstream mechanisms and regulatory function of *SNAC3* in As stress resistance, the expression levels of antioxidant genes (*OsSOD-Cu/Zn, OsCATA*, *OsCATB*, and *OsAPX2*) and stress-related genes (*OsLEA3*, *OsDREB2B*, *OsDREB2A*, *OsSNAC2*, *OsSNAC1*, and *OsCRY1b*) were assessed in the transgenic and non-transgenic rice lines grown under As stress circumstances utilizing qRT-PCR. The results revealed no significant variations in the expression levels of these genes among transgenic and non-transgenic plants grown under normal circumstances (Figure 9A–D; Figure 10A–F). By contrast, under both levels of As stress (25 and 50 µM), the expression levels of the antioxidant genes and five stress-related genes (*OsLEA3*, *OsDREB2B*, *OsDREB2A*, *OsSNAC2*, and *OsSNAC1*) of the two *SNAC3*-OX lines were higher than that of the non-transformed plants, while the two *SNAC3*-RNAi lines indicated remarkably lower levels, comparing to the non-transformed plants (Figure 9A–D; Figure 10A–E). More importantly, under both levels of As stress, the expression level of *OsCRY1b* in the two *SNAC3*-OX lines were considerably lower than those in the non-transgenic plants, while the two *SNAC3*-RNAi lines showed significantly enhanced expression levels of *OsCRY1b*, comparing to the non-transgenic plants (Figure 10F). These findings reveal that *SNAC3* overexpression alleviates As toxicity and associated oxidative damage in rice through inducing genes that mediate free-radical-scavenging pathways and proteins involved in defense mechanisms. Moreover, *SNAC3* overexpression suppressed the expression of *OsCRY1b* (rice *cryptochrome 1b* gene), thereby enhancing As resistance in rice plants. These results were in concordance with those of the enzymic antioxidants assayed in the present study. Our results were also in line with the findings of Yuan et al. [1] and Fang et al. [20], who recorded enhanced stress-responsive gene expression in *ONAC066*- and *SNAC3*-overexpressing lines, respectively, comparing to the non-transgenic stressed-plants. The findings were also in line with those recorded previously by El-Esawi et al. [50] and Cai et al. [51] who revealed that *Rab7*- and *nNOS*-overexpressing rice plants, respectively, showed higher expression levels of abiotic stress-related genes in comparison with the non-transgenic plants grown under stress conditions. Furthermore, our findings agree with the outcomes of Yu et al. [52], who revealed that *OsEm1*-overexpressing rice plants showed increased expression of the late embryogenesis abundant protein (*OsLEA3*) mediating stress resistance, comparing to the non-transgenic plants grown under stress conditions.

### 2.7. Overexpression of SNAC3 Improves While Suppression of SNAC3 Reduces Rice Grain Yield and Yield Components under Arsenic Stress

Environmental stresses influence crop grain yields. To assess the key role of *SNAC3* gene in enhancing rice grain yields, several yield traits, including panicle length, number of spikelets per hill, number of spikelets per panicle, filling rate, number of filled grains per hill, and total grain weight were recorded for the transgenic and non-transgenic plants grown under optimal and As stress treatments. The results revealed no considerable variations in the measured yield parameters among the non-transgenic and transgenic lines grown under optimal circumstances (Table 2). However, under both levels of As stress (25 and 50 µM), all the yield traits of the two *SNAC3*-OX lines were considerably greater than those of the wild-type rice plants, while the two *SNAC3*-RNAi lines showed significantly lower rates of yield parameters, comparing to the non-transgenic plants (Table 2). For instance, the two *SNAC3*-OX lines had a greater filling rate than that of the non-transgenic plants grown under As stress circumstances, leading to significant increments in the total weight of grain. These outcomes demonstrate the key role of *SNAC3* in boosting the grain yield of rice plants grown under As stress conditions. Our findings are in agreement with those of other reports that revealed enhanced grain yields in rice plants overexpressing *OsRab7* [50], *TIFY* [53], *AP37* [54], *OsNRT2.1* [55], and *OsNAC2* [56].

## 3. Materials and Methods

### 3.1. Plant Material and Growth Circumstances

*Oryza sativa* subsp. *japonica* cultivar Giza 177, received from the Egyptian Agricultural Research Center, was employed in all the experiments of the current study. After surface-sterilizing in sodium hypochlorite (0.5%) and washing with H_2_O five times, plant seeds were sown and grown on wet papers for 6 days. Healthy uniform plantlets were chosen, transplanted into plastic containers comprising perlite, peat, and sand (1:1:1, *v*/*v*/*v*), and then left to grow in a growth room at 70% humidity, 300–400 µmol m^−2^ s^−1^ light intensity, 16/8 h light/dark and 27/21 °C. A daily irrigation with a Hoagland nutrient solution [57] was applied to the plants.

### 3.2. Construction of Plasmids and Transformation of Rice Plants

*SNAC3*-overexpression (*SNAC3*-OX) and *SNAC3*-RNAi (*SNAC3*-Ri) constructs were created as stated by Fang et al. [20]. Briefly, to generate the *SNAC3*-OX construct, RNA was taken out from 3-week-old rice cultivar Giza 177 plants utilizing an RNeasy Plant Mini kit (Qiagen, Hilden, Germany), and cDNA was then generated using a Reverse Transcription kit (Qiagen, Hilden, Germany). cDNA of *SNAC3* was then amplified and introduced into the pU1301 vector under *Ubiquitin* promoter control. To create the *SNAC3*-RNAi construct, a *SNAC3*-specific fragment (334 bp) was obtained and inserted into the pDS1301 vector. Obtained constructs were transferred to *Agrobacterium tumefaciens* EHA105, which was then transformed into rice cultivar Giza 117 plants according to *Agrobacterium*-mediated transformation protocol reported by Lin and Zhang [58] with some modifications. Briefly, callus induction was performed on Murashige and Skoog (MS) medium comprising 500 mg/L glutamine, 3.0 mg/l 2,4-dichlorophenoxyacetic acid (2,4-D), 3% sucrose, 500 mg/L proline, and 0.25% phytagel at pH 5.8. Subculture was conducted on MS medium comprising 500 mg/L proline, 2.5 mg/l 2,4-D, 3% maltose, 500 mg/L glutamine, and 0.25% phytagel at pH 5.8. *SNAC3*-OX and *SNAC3*-Ri constructs were created using the primers reported by Fang et al. [20].

### 3.3. Molecular Characterization of Transformed Rice Lines 

Transgenic lines of the T_2_ generation were screened for hygromycin-resistant phenotype segregation on 1/2 MS (Murashige and Skoog) medium supplemented with 50 mg/L hygromycin. Plants exhibiting 3:1 segregation for hygromycin-resistant phenotype were selected as single-copy transformed lines. T_3_ transformed lines exhibiting 100% hygromycin-resistant phenotype on selective medium were chosen as homozygous lines. Homozygous single-copy T_3_ transgenic lines were utilized for all subsequent analyses. Leaves of three-week-old wild-type, *SNAC3*-OX, and *SNAC3*-RNAi plants were harvested for analyzing *SNAC3* expression level utilizing quantitative real-time PCR (qRT-PCR). In brief, total RNA and cDNA were prepared from those collected leaf samples as mentioned above. qRT-PCR analysis was then performed in 5 replications utilizing Qiagen QuantiTect SYBR Green PCR kit in order to analyze *SNAC3* expression level. PCR conditions and *SNAC3*-specific primers were utilized as described by Fang et al. [20]. The housekeeping gene, *Ubiquitin*, was assayed. Relative expression level of *SNAC3* was then calculated as reported by Livak and Schmittgen [59].

### 3.4. Arsenic Stress Treatments

Wild-type, two T_3_ homozygous *SNAC3*-OX (OX-5 and OX-9), and two T_3_ homozygous *SNAC3*-RNAi (Ri-2 and Ri-8) rice lines were used for the As stress treatment experiment. Seeds of these 5 lines were disinfected in sodium hypochlorite (0.5%), washed with H_2_O, and left to grow on wet papers for 6 days. Healthy uniform seedlings were moved into containers comprising peat, sand, and perlite (1:1:1, *v/v/v*). Containers were arranged in a complete randomized design in a growth room at 70% humidity, 27/21 °C, 16/8 h (light/dark), and a light intensity of 250–300 µmoL m^−2^ s^−1^. The plants received daily irrigation with a Hoagland nutrient solution for 3 weeks. The 27-day-old plants were assigned to groups representing different treatments as follows: (i) control plants irrigated with a Hoagland nutrient solution; (ii) As-stressed plants irrigated with a Hoagland solution containing 25 µM As; and (iii) As-stressed plants irrigated with a Hoagland solution containing 50 µM As. As concentrations were prepared using sodium arsenite (NaAsO_2_) and selected based on our preliminary standardization experiments and previous rice studies [60,61]. After 12 days of As stress treatments, some plant samples were harvested for use in physio-biochemical and molecular assays. Following a 10-day recovery period, the survival rate of plants was estimated.

To estimate the plant yield traits under As stress circumstances, the wild-type and T_3_ transformed rice plants were moved into larger containers filled with natural paddy soil. Containers were placed in a complete randomized design in growth rooms at 27/21 °C. Plants were fed daily with a Hoagland solution. Approximately 12–14 days before panicle heading stage, plants were assigned to different treatment groups and irrigated with Hoagland nutrient solutions containing 0 (control), 25 µM As, or 50 µM As. All these treatments continued for 24 days, then the plants were grown at standard growth conditions till harvesting. At maturity stage, the yield traits such as total number of spikelets per hill, panicle length (cm), number of spikelets per panicle, total grain weight (g), filling rate (%), and number of filled grains per hill were estimated.

### 3.5. Estimating the Morphological Characteristics and Relative Water Content 

The plant samples were collected and washed using distilled water. A measuring scale was utilized for determining the plant height. The root and shoot samples were then separated. Fresh weights of shoots and roots were estimated. Dry weights of shoots and roots were also estimated after exposure to 70 °C for 48 h. Moreover, the methodology of Yamasaki and Dillenburg [62] was adopted to calculate the leaf relative water content (RWC).

### 3.6. Estimating Arsenic Content in Plant Roots and Leaves

As concentration were assessed in plant roots and leaves as previously stated by Mousavi et al. [61] with slight adjustments. Briefly, root and leaf tissue samples were cleaned with water and then oven-dried at 70 °C for 72 h. Dried tissue samples were mixed with a solution of HNO_3_:H_2_O_2_ (1:4 ratio). As concentration was then measured using ICP–mass spectrometry (Optima 7900DV, PerkinElmer, MA, USA).

### 3.7. Measurement of Oxidative Stress Biomarkers

Estimation of the leaf content of hydrogen peroxide (H_2_O_2_) was carried out using the methodology indicated by Velikova et al. [63] with slight adjustments. In brief, 0.5 g of fresh leafy sample was dissolved in 5 mL of trichloroacetic acid (0.1 %) and then centrifuged at 10,000× *g* for 20 min. A mixture of supernatant (0.5 mL), 10 mM potassium phosphate buffer (0.5 mL) and 1 M KI (1 mL) was prepared and then vortexed before measuring the absorbance at 390 nm. H_2_O_2_ concentration was calculated utilizing H_2_O_2_ standard curve.

Production of leaf malondialdehyde (MDA) was estimated using the methodology of Heath and Packer [64] with slight modifications. Leafy samples (1 g) were homogenized in TCA (20 mL, 0.1%), followed by a 7-min centrifugation at 14,000× *g*. A mixture of supernatant (1.5 mL) and thiobarbituric acid (6.0 mL, 0.5%) in TCA (20%) was prepared and then agitated at 95 °C for 25 min. This was followed by chilling on ice and a 10-min centrifugation at the highest speed. Absorbance of supernatants was monitored at 532 and 660 nm. Moreover, the methodology of Dionisio-Sese and Tobita [65] was adopted to estimate leaf electrolyte leakage (*EL*).

### 3.8. Estimation of Total Chlorophyll, Gas-Exchange Parameters, Soluble Protein, Sugars, Proline, Phenolics, and Flavonoids

Estimation of total leaf chlorophyll was done as indicated by Lichtenthaler [66]. In brief, maceration of fresh leaves (of 0.2 g) was performed in acetone (50 mL, 80%). The extract was centrifuged at 14,000× *g* for 8 min. Supernatant absorbance was measured at 663 and 645 nm. Moreover, estimation of net photosynthesis rate (*P_n_*), stomatal conductance (*g_s_*), and transpiration rate (*E*) was performed on the leaf midrib in the early morning following the protocol reported by Holá et al. [67].

To determine the total protein content of leaves, samples of fresh leaves were macerated in Tris buffer (100 mM, pH 8.0) using a cold mortar. The extracts were exposed to a 15-min centrifugation at 20,000× *g*. Bradford assay [68] was then utilized to determine total protein content. Total soluble sugar of leaves was also spectrophotometrically estimated at 485 nm following the reported methodology of Dey [69].

Determination of proline level was performed as indicated by Bates et al. [70] with slight adjustments. Briefly, 0.5 g of leaf sample was macerated in 3 mL of 5% (*w*/*v*) sulfosalicylic acid, followed by centrifugation at 9000× *g* for 8 min. Supernatant (0.5 mL) was diluted with sterile H_2_O (1 mL) and mixed with 2% ninhydrin (2 volumes) by a vortex mixer. This was followed by a 35-min incubation at 95 °C and then cooling. An equivalent amount of toluene was added into the solution, and the absorbance was measured at 520 nm for the upper aqueous layer using a spectrophotometer. The L-proline calibration curve was utilized as a standard for calculation.

The concentration of total phenolics in plant leaves was estimated through the homogenization of leafy samples (2 g) in 80% of methanol (10 mL), followed by heating up at 70 °C for 20 min. A volume of extract (2 mL) was diluted with H_2_O (10 mL) containing 1 N Folin–Ciocalteau reagent (500 μL), followed by incubation at 30 °C. To estimate the contents of phenols, the mixture absorbance was read at 725 nm [71]. Gallic acid was utilized as a standard. 

The content of total flavonoids in plant leaves was also estimated according to Zhishen et al. [72]. Dried leaf powder (1 g) was dissolved in sterilized H_2_O (100 mL), followed by filtration of the extract and mixing with AlCl_3_, distilled H_2_O, and NaNO_2_ solution. Then, NaOH solution was transferred to the mixture, followed by dilution with distilled H_2_O. The absorbance was monitored at 510 nm. The catechin standard curve was utilized.

### 3.9. Antioxidant Enzyme Assays

Approximately 1 gm of leaf sample was macerated in 100 mM Tris-HCl (pH 7.5) combined with 10 mM MgCl_2_, PVP-40 (1.5%), 5 mM Dithiothreitol, 1 mM EDTA, 1 mM phenylmethanesulfonyl fluoride, 5 mM magnesium acetate, and 1 μg.mL^−1^ aproptinin. The suspension was refined, followed by a 10-min centrifugation at 11,000 rpm. Enzyme activities were monitored using the supernatant. For APX activity analysis, 2 mM AsA was utilized in the homogenization of leaf sample.

Ascorbate peroxidase (APX) level in the plant leaf was estimated following the methodology of Nakano and Asada [73], and the optical density was monitored at 265 nm using a spectrophotometer. The activity of APX was expressed in enzyme unit per gram fresh weight (U/g.FW). Superoxide dismutase (SOD) activity was recorded following the methodology of Kono [74] and according to nitroblue tetrazolium photoreduction. A spectrophotometer was used to record the absorbance at 540 nm. Moreover, determination of catalase (CAT) activity was performed following the reported methodology of Aebi [75]. Absorbance was monitored at 240 nm. Peroxidase (POD) enzyme level was also assessed according to Putter and Becker [76]. Production rate of oxidized guaiacol was read at 436 nm. The activity of POD, CAT and SOD was also expressed as U/g.FW.

### 3.10. Analysis of Stress-Tolerant Genes Expression

Expression of antioxidant genes (*OsSOD-Cu/Zn*, *OsCATB*, *OsCATA*, and *OsAPX2*) and 6 abiotic stress-associated genes (*OsLEA3*, *OsDREB2B, OsDREB2A*, *OsSNAC2*, *OsSNAC1*, and *OsCRY1b*) were measured in the transformed and non-transformed plants under As stress conditions. In brief, total RNA and cDNA were isolated from the harvested leaf samples as mentioned above. qRT-PCR analysis was then performed utilizing the Qiagen QuantiTect SYBR Green PCR kit. PCR circumstances were set up as outlined by Vighi et al. [77] and Cai et al. [51]. A set of genes-specific primers [51,52,77,78] were applied for amplification. An internal reference gene, *UBQ10*, was used [77]. The relative expression levels of genes were calculated as reported by Livak and Schmittgen [59].

### 3.11. Statistical Analysis

One-way analysis of variance and Duncan’s multiple range test were applied to analyze the obtained data. Values indicate means ± standard error (SE), and are significantly different at *p* ≤ 0.05.

## 4. Conclusions

Arsenic toxicity adversely restricts rice growth and yield. The crucial function of *SNAC3* in boosting As stress resistance and grain yield in rice has been investigated. Two *SNAC3*-OX lines and two *SNAC3*-RNAi lines were created and subjected to As stress treatments. *SNAC3* overexpression significantly augmented rice tolerance to As stress and boosted grain yield, while *SNAC3* suppression increased rice sensitivity to As and reduced grain yield. *SNAC3* enhanced As stress resistance and grain productivity in rice through modulating antioxidant machinery, osmolyte synthesis, and stress-responsive genes, and could be a useful prospect for further improvement of crop stress resistance and grain productivity. Further analysis might be carried out to unravel the regulatory network of *SNAC3* in greater depth and to further dissect the molecular mechanisms underlying stress resistance in rice.

## Figures and Tables

**Table 1:** Morphological parameters of the wild type (WT) and transformed plants under varying doses of arsenic stress for 12 days.

Treatment As (µM)	Rice Line	Plant Height (cm)	Root Fresh Weight (mg)	Root Dry Weight (mg)	Shoot Fresh Weight (mg)	Shoot Dry Weight (mg)
Normal (0)	WT	28.2 ± 0.87 a	8.9 ± 0.30 a	1.5 ± 0.17 a	24.8 ± 0.49 a	4.9 ± 0.27 a
	OX-5	28.4 ± 0.88 a	9.0 ± 0.29 a	1.7 ± 0.13 a	25.1 ± 0.46 a	5.1 ± 0.32 a
	OX-9	28.3 ± 0.85 a	9.1 ± 0.34 a	1.6 ± 0.14 a	25.0 ± 0.45 a	5.0 ± 0.27 a
	Ri-2	28.2 ± 0.84 a	8.8 ± 0.31 a	1.5 ± 0.15 a	24.7 ± 0.48 a	5.0 ± 0.28 a
	Ri-8	28.1 ± 0.81 a	8.7 ± 0.30 a	1.6 ± 0.14 a	24.8 ± 0.47 a	4.8 ± 0.26 a
25	WT	20.1 ± 0.91 e	5.0 ± 0.33 b	0.8 ± 0.13 c	15.9 ± 0.48 d	3.1 ± 0.29 c
	OX-5	26.5 ± 0.77 b	8.7 ± 0.28 a	1.1 ± 0.13 b	22.4 ± 0.61 b	4.1 ± 0.32 b
	OX-9	26.2 ± 0.81 b	8.6 ± 0.31 a	1.1 ± 0.16 b	22.1 ± 0.58 b	4.0 ± 0.25 b
	Ri-2	17.2 ± 0.83 f	3.2 ± 0.32 c	0.5 ± 0.14 d	11.9 ± 0.51 e	2.3 ± 0.23 d
	Ri-8	17.3 ± 0.84 f	3.3 ± 0.30 c	0.5 ± 0.12 d	11.8 ± 0.55 e	2.4 ± 0.24 d
50	WT	16.1 ± 0.74 g	3.5 ± 0.32 c	0.6 ± 0.10 d	12.1 ± 0.44 e	2.5 ± 0.21 d
	OX-5	23.9 ± 0.91 c	4.8 ± 0.33 b	0.8 ± 0.15 c	19.0 ± 0.48 c	3.4 ± 0.31 c
	OX-9	21.9 ± 0.88 d	4.6 ± 0.29 b	0.8 ± 0.14 c	18.7 ± 0.52 c	3.3 ± 0.29 c
	Ri-2	13.4 ± 0.83 h	2.1 ± 0.29 d	0.3 ± 0.15 e	08.1 ± 0.48 f	1.7 ± 0.27 e
	Ri-8	13.8 ± 0.81 h	2.2 ± 0.27 d	0.3 ± 0.14 e	08.0 ± 0.52 f	1.6 ± 0.30 e

Value represent means ± SE (*n* = 5). Under the same circumstances, means with different letters are significantly different (*p* ≤ 0.05).

**Table 2:** Yield traits of the wild-type (WT) and transgenic rice lines subjected to normal and arsenic stress circumstances for 12 days.

Treatment As (µM)	Rice Line	Panicle Length (cm)	Number of Spikelets per Panicle	Total Number of Spikelets per Hill	Number of Filled Grains Per Hill	Filling Rate (%)	Total Grain Weight (g)
Normal (0)	WT	18.0 ± 1.08 a	90.0 ± 7.98 a	1190.7 ± 144.2 a	1047.1 ± 121.2 a	88.1 ± 11.42 a	17.9 ± 1.22 a
	OX-5	18.1 ± 1.12 a	91.0 ± 8.03 a	1186.4 ± 138.3 a	1049.4 ± 122.5 a	88.5 ± 12.11 a	18.0 ± 1.30 a
	OX-9	18.0 ± 1.16 a	89.5 ± 9.13 a	1182.5 ± 132.4 a	1052.3 ± 124.2 a	89.4 ± 10.55 a	18.1 ± 1.41 a
	Ri-2	18.1 ± 1.15 a	89.2 ± 9.11 a	1184.7 ± 141.6 a	1051.4 ± 121.6 a	88.9 ± 10.73 a	17.9 ± 1.32 a
	Ri-8	17.9 ± 1.14 a	91.1 ± 9.11 a	1193.1 ± 141.1 a	1043.9 ± 131.3 a	89.8 ± 11.39 a	17.8 ± 1.21 a
25	WT	14.0 ± 1.21 c	80.2 ± 8.11 b	1095.4 ± 131.2 c	799.2 ± 128.9 d	70.2 ± 11.21 d	14.1 ± 1.32 c
	OX-5	17.9 ± 1.30 a	89.3 ± 8.36 a	1181.1 ± 140.7 a	984.4 ± 135.1 b	82.3 ± 11.35 b	17.6 ± 1.28 a
	OX-9	17.8 ± 1.25 a	88.4 ± 8.12 a	1179.3 ± 134.8 a	978.6 ± 133.7 b	81.7 ± 10.29 b	17.8 ± 1.26 a
	Ri-2	10.2 ± 1.13 e	71.4 ± 7.88 c	1017.5 ± 132.4 d	711.5 ± 127.6 e	62.1 ± 11.33 e	11.2 ± 1.17 e
	Ri-8	10.4 ± 1.15 e	73.6 ± 7.69 c	1021.7 ± 126.4 d	706.8 ± 124.2 e	61.8 ± 11.45 e	11.1 ± 1.13 e
50	WT	12.1 ± 1.13 d	66.3 ± 8.10 d	1021.5 ± 141.2 d	621.8 ± 121.3 f	54.7 ± 14.33 f	12.7 ± 1.17 d
	OX-5	16.1 ± 1.25 b	79.3 ± 8.12 b	1139.3 ± 127.6 b	903.4 ± 122.4 c	75.1 ± 14.38 c	15.8 ± 1.21 b
	OX-9	15.9 ± 1.31 b	80.1 ± 8.56 b	1142.5 ± 131.2 b	897.8 ± 113.5 c	75.8 ± 12.47 c	15.2 ± 1.32 b
	Ri-2	08.9 ± 1.32 f	53.7 ± 8.51 e	0944.8 ± 132.4 e	535.3 ± 122.3 g	43.4 ± 14.19 g	10.1 ± 1.31 f
	Ri-8	09.1 ± 1.36 f	54.1 ± 8.56 e	0950.4 ± 132.2 e	540.3 ± 125.8 g	42.6 ± 14.21 g	10.2 ± 1.34 f

Values represent means ± SE (*n* = 5). Under the same circumstances, means with different letters are significantly different (*p* ≤ 0.05).

## Data Availability

All the data of the present study are included in this published article.
